# Efficient CRISPR/Cas9-based genome editing in carrot cells

**DOI:** 10.1007/s00299-018-2252-2

**Published:** 2018-01-13

**Authors:** Magdalena Klimek-Chodacka, Tomasz Oleszkiewicz, Levi G. Lowder, Yiping Qi, Rafal Baranski

**Affiliations:** 1 Institute of Plant Biology and Biotechnology, Faculty of Biotechnology and Horticulture, University of Agriculture in Krakow, AL. 29 Listopada 54, 31-425 Krakow, Poland; 2 Department of Biology, East Carolina University, N108 Howell Science Complex, Greenville, NC 27858 USA; 3 Department of Plant Science and Landscape Architecture, University of Maryland, 4291 Fieldhouse Dr, College Park, MD 20742 USA; 4 Institute for Bioscience and Biotechnology Research, University of Maryland, 9600 Gudelsky Dr, Rockville, MD 20850 USA

**Keywords:** *Daucus carota*, Gene knockout, Genome editing, Flavanone 3-hydroxylase, Site-directed mutagenesis

## Abstract

**Key message:**

The first report presenting successful and efficient carrot genome editing using CRISPR/Cas9 system.

**Abstract:**

Clustered Regularly Interspaced Short Palindromic Repeats (CRISPR)/CRISPR-associated (Cas9) is a powerful genome editing tool that has been widely adopted in model organisms recently, but has not been used in carrot—a model species for in vitro culture studies and an important health-promoting crop grown worldwide. In this study, for the first time, we report application of the CRISPR/Cas9 system for efficient targeted mutagenesis of the carrot genome. Multiplexing CRISPR/Cas9 vectors expressing two single-guide RNA (gRNAs) targeting the carrot flavanone-3-hydroxylase (*F3H*) gene were tested for blockage of the anthocyanin biosynthesis in a model purple-colored callus using *Agrobacterium*-mediated genetic transformation. This approach allowed fast and visual comparison of three codon-optimized Cas9 genes and revealed that the most efficient one in generating *F3H* mutants was the *Arabidopsis* codon-optimized AteCas9 gene with up to 90% efficiency. Knockout of *F3H* gene resulted in the discoloration of calli, validating the functional role of this gene in the anthocyanin biosynthesis in carrot as well as providing a visual marker for screening successfully edited events. Most resulting mutations were small Indels, but long chromosome fragment deletions of 116–119 nt were also generated with simultaneous cleavage mediated by two gRNAs. The results demonstrate successful site-directed mutagenesis in carrot with CRISPR/Cas9 and the usefulness of a model callus culture to validate genome editing systems. Given that the carrot genome has been sequenced recently, our timely study sheds light on the promising application of genome editing tools for boosting basic and translational research in this important vegetable crop.

**Electronic supplementary material:**

The online version of this article (10.1007/s00299-018-2252-2) contains supplementary material, which is available to authorized users.

## Introduction

Clustered regularly interspaced short palindromic repeats (CRISPR) together with CRISPR-associated protein (Cas) act as an adaptive immune system against phages and are widely distributed among bacteria and archaea (Barrangou and Marraffini 2014; Makarova et al. 2015). The CRISPR locus contains information about the previous bacteriophage or plasmid infections due to acquiring short fragments of their DNA as spacers when new invading DNA is recognized (Barrangou et al. 2007). This native defense system consists of a protein complex with nucleolytic activity and two RNA molecules, crRNA located in the CRISPR locus, and tracrRNA partially complementary to crRNA (Jinek et al. 2012). The number and function of proteins in the nucleolytic complex depends on species. In *Staphylococcus pyogenes*, a single Cas9 enzyme with two nuclease domains, RuvC and HNH, cleaves the non-target and target DNA strands, respectively (Makarova et al. 2015). Recognition of a specific viral sequence by a Cas9–RNA complex requires the presence of a protospacer adjacent motif (PAM) in a foreign DNA located at the 3′ end of the target site. PAM is a short, usually 3–5 nt sequence characteristic for a given microbial species (Anders et al. 2014). The CRISPR immune system has been repurposed for genome editing. Current editing systems predominantly utilize *S. pyogenes* Cas9 protein, which requires the NGG PAM sequence at target DNA for correct site recognition. The crRNA and tracrRNA sequences can be combined into a single guide RNA molecule (Jinek et al. 2012), which subsequently enhanced its application in eukaryotic cell genome editing. The Cas9 enzyme causes DNA double-strand break at the target locus, which is then repaired by the host cell repair system. Non-homologous end-joining (NHEJ) is a dominant repair pathway often leading to imperfect repair, generating mutations (Belhaj et al. 2013). After the first demonstration of human genome editing using the CRISPR/Cas9 (Cong et al. 2013; Mali et al. 2013), the CRISPR/Cas9 system has been extensively and effectively employed for genome edition in human cells, mice, and other species, including plants (Andersson et al. 2017; Braatz et al. 2017; Meng et al. 2017).

Anthocyanins are a large group of over 500 pigments abundant in plant kingdom accumulated in the cell vacuole of some organs. Variation of substrate and possible side-group arrangements determines light absorbance spectrum of anthocyanin compounds; thus, their color can vary widely from purple, blue to red (Mazza and Miniati 1993). The occurrence of these pigments in plant tissue is the result of a multistep and branched flavonoid biosynthesis pathway. A colorless naringenin (flavanone) is a precursor in the upstream pathway. It is converted to dihydroflavonols, which are then converted to unstable pigments, anthocyanidins. Anthocyanins, which are stable compounds, are glycosylated and methylated derivatives of anthocyanidins. Thus, accumulation of anthocyanin pigments requires colorless dihydroflavonols to be synthesized first. The later compounds, namely dihydrokaempferol, dihydroquercetin, and dihydromyricetin, are products of naringenin hydroxylation via three branches of the pathway, and three hydroxylases may be involved in their synthesis depending on the pathway route. Flavanone-3-hydroxylase (F3H) attaches a hydroxy group to carbon 3 in the flavanone phenyl C-ring of the substrate compound that is a characteristic side group of anthocyanins, and F3H is the only enzyme that catalyses this hydroxylation reaction in all three routes. Thus, F3H is considered to be critical for anthocyanin biosynthesis (Cheynier et al. 2013; Petrussa et al. 2013). When expression of F3H is inhibited, the synthesis of these pigments is restricted as demonstrated in orange carnation (Zuker et al. 2002). Carrot (*Daucus carota* L. var. *sativus* Hoffm.) is able to synthesize mainly cyanidin-based anthocyanins (Kammerer et al. 2004). This group of pigments has purple color whose stability, saturation and hue depend on the type of glycosylation and acylation reactions, cell pH, and are modified by environmental factors (Stintzing et al. 2002; Kramer et al. 2012). Some cultivars, traditionally grown in Asia and Middle East, develop purple storage root and accumulate a large amount of anthocyanins up to 17,414 mg/kg dry weight (Kammerer et al. 2004). High pigment content was correlated with expression of the flavonoid pathway key genes, including the *F3H* gene (Yildiz et al. 2013; Xu et al. 2014). In contrast, carrots developing orange storage roots may accumulate only very small amount of anthocyanins in hypocotyl, basal part of petioles, and in the skin of storage root shoulders (Leja et al. 2013). However, visible purple color in these tissues is an evidence of functional anthocyanin biosynthesis pathway.

Carrot is a highly valuable vegetable crop uniquely accumulating pro-vitamin A carotenoids in its storage root and has been used as a model species in biotechnology for decades. In fact, it was one of a few species utilized for the development of plant tissue culture techniques including callus induction and its maintenance in vitro. Carrot is also amenable to genetic transformation using *Agrobacterium* (Baranski 2008). However, progress in carrot genetics has been much slower than in many other crop species mainly due to biennial reproduction cycle, outcrossing, and high inbreeding depression effect (Rubatzky et al. 1999). Callus tissue cultured in vitro may be considered as a valuable alternative to plant requiring long cultivation while implemented for basic research at cellular level, including genetic engineering (Carciofi et al. 2012; Tran and Sanan-Mishra 2015). Carrot callus grows fast, which can serve as a convenient protoplast source or be easily stimulated for embryogenesis allowing massive clonal propagation. Thus, it is a feasible material for genetic and genomic research at cell and tissue levels, and its maintenance is much less laborious than the use of cell suspension culture which requires frequent renewals (Baranski 2008).

Recent publication of the genome sequencing data of a doubled haploid carrot line has opened new perspectives for crop improvement (Iorizzo et al. 2016). To boost progress in carrot research, we sought to apply, for the first time, the CRISPR/Cas9 system in carrot cells and to optimize it for further gene editing purposes. We have established a unique callus culture characterized by stable deep purple color due to anthocyanin pigments accumulation. In this study, we conveniently take advantage of this model callus tissue to demonstrate CRISPR/Cas9 genome editing in carrot. We targeted *F3H*, a key gene in the anthocyanin biosynthesis pathway, using multiplexing CRISPR/Cas9 vectors. *F3H* was functionally validated in carrot cells as loss of function due to CRISPR editing resulting in white calli, suggesting blockage of the anthocyanin biosynthesis pathway. Using this fast and easy assay, we compared three CRISPR/Cas9 systems differing in codon-optimized Cas9 genes and identified the most efficient CRISPR/Cas9 for carrot genome editing.

## Materials and methods

### Plant material

We used a carrot callus line characterized by an intensive purple color due to anthocyanin accumulation. White callus line from which the purple callus was derived was also used as the reference for anthocyanin content analysis. Callus was maintained in Petri dishes with a phytagel solidified Gamborg B5 salts with vitamins medium (Duchefa, Haarlem, The Netherlands) supplemented with 1 mg/L 2,4-dichlorophenoxyacetic acid (2,4-D, Sigma, St. Louis, USA), 0.025 mg/L kinetin (Duchefa, Haarlem, The Netherlands), and 30 g/L sucrose; pH 5.8. Callus was cultured at 26 °C in the dark and was subcultured every 3 weeks to a fresh medium.

### Vector construction

CRISPR/Cas9 vectors for multiplex gene targeting were developed according to Lowder et al. (2015) and then transferred to *Agrobacterium tumefaciens* strain LBA 4404 by electroporation (Main et al. 1995). Vector T-DNA contained at its right border a *Cas*9 gene driven by the 2 × 35S CaMV promoter followed by two single guide RNAs, each controlled by the AtU3 promoter. The left T-DNA fragment contained the hygromycin resistance *aph* gene with 35S CaMV promoter. Three Cas9 entry clones (pYPQ154, pYPQ166, and pYPQ167) containing three codon-optimization variants of SpCas9 genes were used: AteCas9 (Fauser et al. 2014; Schiml et al. 2014), zCas9 (Xing et al. 2014) and Cas9p (Ma et al. 2015). Two gRNAs, gRNA3—ATTAGAGCCCGGGACTACT and gRNA4—AAGTTTTGTCAGAGGCCAT, were designed to target the second exon of the *F3H* gene at sites adjacent to CGG-PAM and GGG-PAM, respectively, based on the sequence of the reference *F3H* gene (NCBI Acc. no. XM_017385173) deposited within a doubled haploid DH1 carrot genome sequencing project (Iorizzo et al. 2016).

### *Agrobacterium-mediated* callus transformation

*Agrobacterium tumefaciens* was growing in lysogeny broth supplemented with 50 mg/L of kanamycin at 26 °C and using a rotary shaker with 260 rpm. An overnight culture was centrifuged at 6000 rpm for 10 min. The pellet was resuspended in callus culture liquid medium with addition of 100 µM acetosyringone (Sigma, St. Louis, USA) and diluted to OD_600_ = 0.5. Two-week-old callus clumps of about 5 mm diameter were placed in a Petri dish and immersed in *A. tumefaciens* inoculum for 20 min. Then, clumps were blotted on a sterile filter paper and placed on a solidified culture medium, followed by 3-day co-cultivation at 26 °C in the dark. Subsequently, for *Agrobacterium* elimination, calli were rinsed with sterile water containing 800 mg/L cefotaxime and 400 mg/L timentin, and were transferred to a fresh callus culture medium supplemented with 400 mg/L cefotaxime and 200 mg/L timentin. After 4 weeks, callus clumps were transferred and spread on a fresh medium supplemented with 25 mg/L hygromycin. Single, small new callus clumps developing after a 4-week selection were picked up and transferred periodically to the same selection medium until the end of the experiments.

### PCR and restriction fragment analysis

Genomic DNA was extracted from callus using a CTAB method described by Rogers and Bendich (1988) with modifications. Small fragments of transgenic callus were placed in 2 ml eppendorf tubes with 100 µl of CTAB buffer and two 3 mm beads, and then grinded in the Retsch Mixer Mill MM400 (Retsch GmbH, Haan, Germany) for 3 min at room temperature. PCR was performed using the Eppendorf Mastercycler thermocycler (Eppendorf, Hamburg, Germany) and primers listed in Table 1, and the reaction was set up in a volume of 10 µl containing 0.1 µM of each primer, and 2 × buffer including *Taq* polymerase and dNTPs (PCR Mix Plus, A&A Biotechnology, Gdynia, Poland). The PCR conditions were as follows: the initial denaturation at 94 °C for 4 min, 35 cycles of 45 s at 94 °C, 30 s at 56 °C or 60 °C (depending on primers used), and 60 s at 72 °C, and the final extension for 5 min at 72 °C. PCR products amplified with F3H primers were 100 × diluted and used as templates for the next PCR round of a nested PCR. Final PCR products were digested with *Nco*I High-Fidelity enzyme (NEB R3193S, Ipswich, USA) 37 °C for 3 h. PCR products and restricted fragments were visualized using MidoriGreen Advance (Nippon Genetics) after electrophoresis in 1% agarose (Prona Plus, Conda, Spain) gel.

**Table 1:** Primers used for PCR and Sanger sequencing

Name	Sequence 5′–3′	Annealing temp. (°C)	Expected product length (bp)	Amplified fragment
F3H_FO	GAGAAACTCCGGTTCGATATG	56	709	*F3H*; 1st step in nested PCR
F3H_RO	CTGAACAGTGATCCAGGTTT			
F3H_FM	CGTGTTATCGTTGGGATCGG	56	538	*F3H*; 2nd step in nested PCR
F3H_RM	AGCAAGAGCGTAATTGTGCC			
35S-Cf3	CCACGTCTTCAAAGCAAGTGG	60	AteCas9: 576, 903 zCas9: 695, 1022 Cas9p: 624, 951	35S:*Cas9*
Cas9-R	TTGGGTGTCTCTCGTGCTTC			
Aph-F	AAGGAATCGGTCAATACACTACATGG	60	398	*aph*
Aph-R	AAGACCAATGCGGAGCATATACG			
F3H_FI	ATCACTTTAAAAAGGTTATCAGGG	56	–	Used for sequencing

Undigested fragments were eluted from the gel and purified using the Promega Wizard^®^ SV gel and PCR clean-up system, cloned into pGEM plasmids (Promega, Madison, USA) after genetic transformation to *E. coli*, plasmids were isolated using GeneJET Plasmid Miniprep Kit (Thermo Scientific, Waltham, USA) according to the manufacturer’s instruction. Samples were subjected to Sanger sequencing with F3H_FI primer (Genomed, Warsaw, Poland). Reads were manually aligned to the reference sequence using the BioEdit software.

### Anthocyanin content

Anthocyanin content was determined using a modified pH differential method (Lee et al. 2005). Small callus clumps were homogenized in the extraction buffer (70% ethanol; v/v, acidified with 0.1% 1N HCl; v/v) and filled up to 1 ml. Homegenized tissue was vortexed and shaked for 15 min for complete anthocyanin extraction. To remove any cell debris, extracts were centrifuged for 10 min at 14,000 rpm. Acquired extracts were diluted in pH 1.0 (50 mM KCl) and pH 4.5 (400 mM sodium acetate) buffers and vortexed. Five independent extractions were performed for each callus line. Absorbance of each sample at 520 and 700 nm was measured using the NanoDrop™ 2000c spectrophotometer (Thermo Scientific, Waltham, USA). Anthocyanin content was calculated based on molar weight (449.39) and molar extinction coefficient (26,900) of cyanidin-3-glucoside. Results are presented in mg of cyanidin-3-glucoside (C3G) equivalent per 100 g of callus tissue.

### Protoplast isolation and microscopy

Protoplasts from small callus clumps were isolated according to the protocol previously described (Grzebelus et al. 2012). Due to low amount of tissue available for isolation, volumes of used solutions were reduced eight times. Purified protoplasts were resuspended in 30 µl of the protoplast culture medium. A drop of medium containing protoplasts was placed on a microscopic slide and observed under bright-field microscope (Zeiss Axiovert S100, obj. 10 ×). White and purple protoplasts were counted in five vision fields. Proportions of calli and protoplasts differing in color were compared using statistical tests for significance level between two proportions and Chi-square, respectively.

## Results

### Cas9 variants targeting the *F3H* gene generate phenotypic variation but with different frequencies

**Fig. 1:**
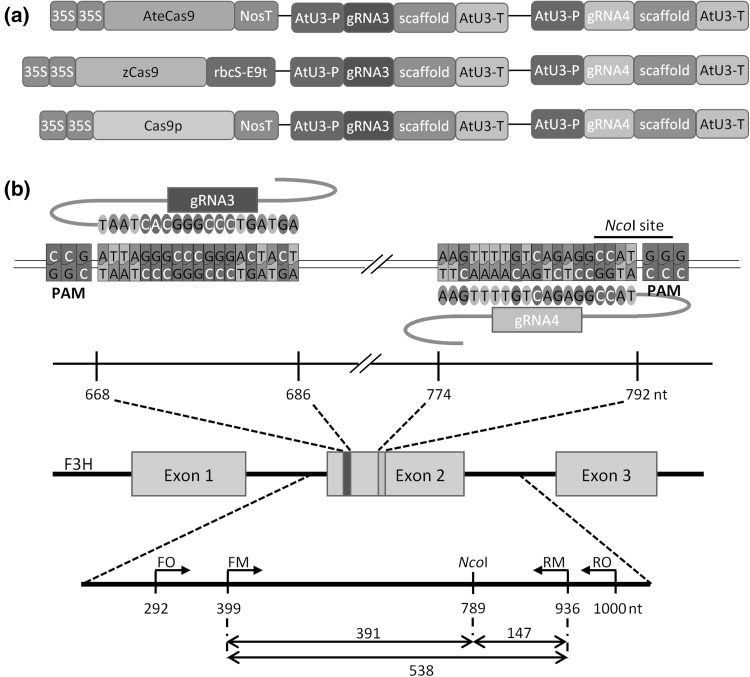
Schematic representation of the *F3H* carrot gene targeted by Cas9/gRNA complexes. Vectors used for *A. tumefaciens*-mediated transformation of purple-colored callus differ only by sequences of codon-optimized *SpCas9* sequences followed by terminator sequences. Target sites of gRNA3 *in blue* and gRNA4 *in pink* are shown relative to PAM sequences in exon2 of the target *F3H* gene. Upper scale shows positions of sequences targeted by gRNAs. Lower scale shows positions of PCR primers and *Nco*I cleavage site together with expected lengths of the PCR amplicon and restriction fragments. Nucleotide positions on both scales refer to the reference *F3H* sequence (NCBI no. XM_017385173) in DH1 carrot. A nucleotide mismatch between gRNA3 and the *F3H* sequence in purple callus is indicated in the gRNA3 sequence *in red. 35S* CaMV 35S promoter, *NosT* nopaline synthase terminator, *rbcS-E9t* terminator of the ribulose-1,5-bisphosphate carboxylase/oxygenase small subunit E9 gene, *AtU3-P* and AtU3-T, *A. thaliana* U3 promoter and terminator, respectively, gRNA3 and gRNA4, guide RNA sequences complementary to the *F3H* carrot gene target sites. **a** Three fragments of vector T-DNA with codon-optimized *SpCas9* genes and two gRNAs used for callus transformation. **b** Concept design of gRNAs targeting the *F3H* gene and expected PCR and restriction fragments

The utilization of CRISPR/Cas9 for plant genome editing is very promising in both basic and applied research. This system, however, has not been used in carrot before. Therefore, we intended to validate the system as well as to develop a model that can be easily and efficiently used in further research enabling carrot genome editing. We first wanted to verify that CRISPR/Cas9 system is able to induce mutations in the carrot genome. For this purpose, we defined a model system that consisted of a carrot callus accumulating a high level of anthocyanins. We chose to compare three SpCas9 vectors differing in codon-optimization (Fig. 1a). We hypothesized that Cas9 can be used to induce mutations in the *F3H* gene leading to blockage of the anthocyanin biosynthesis pathway and resulting in discolored callus cells as compared to the original purple callus cells that contain functional *F3H*. To validate this approach, we designed two gRNAs targeting the *F3H* exon2 (Fig. 1b). After callus transformation, new hygromycin resistant calli had started to develop within 4 weeks of culture, and then, they were selected for further growth. The presence of introduced *aph* and *Cas9* genes in these calli was confirmed by PCR (Supplementary Fig. S1), suggesting successful transformation.

**Fig. 2:**
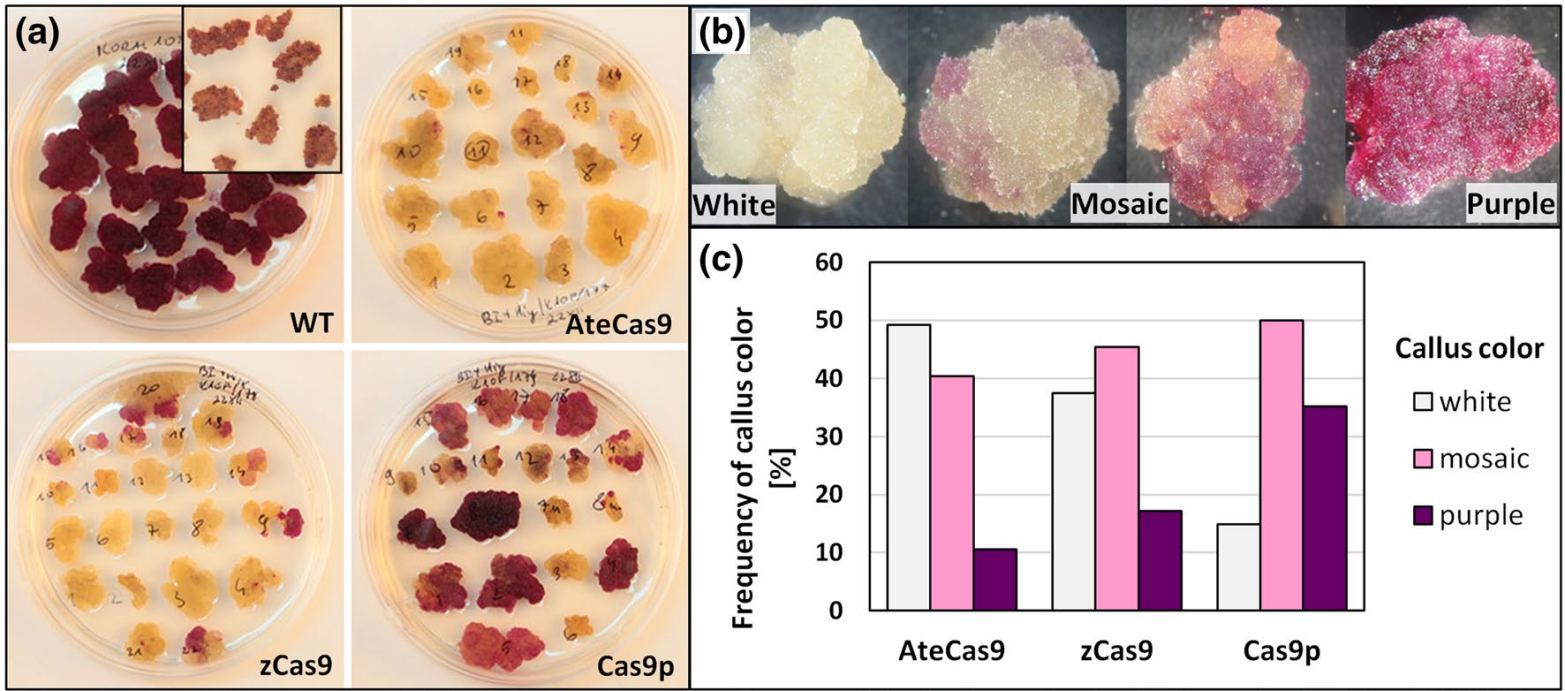
Visual identification of mutant purple carrot callus after *A.tumefaciens*-mediated transformation with three codon-optiomized *SpCas9* genes. **a** Petri dishes with transgenic decolored callus after selection on hygromycin. Upper left Petri dish with wild-type purple callus growing on medium without antibiotic. Inset—WT callus exposed to hygromycin. **b** Individual transgenic calli differing in degree of discoloration from white, mosaic to purple. **c** Frequency of callus of different color identified after selection on hygromycin medium depending on the SpCas9 system used

The developing calli clearly exhibited color variation. Some calli retained deep purple color similar to a non-transformed wild-type (WT) calli, while others were completely white (Fig. 2a). Several calli had mosaic color with white, light purple, or dark purple parts (Fig. 2b). In general, the color of individual calli did not change during the whole culture process. In contrast, WT purple carrot calli grew continuously with deep purple color during the whole experiment when maintained on hygromycin-free medium, while on the selection medium, it did not grow, turned brown, and eventually died due to sensitivity to hygromycin.

Interestingly, we found the frequency of calli differing in color depended on the delivered vector constructs. Most calli developed after transformation with the *Arabidopsis* codon-optimized AteCas9 gene were discolored or showed considerably lighter pigmentation (Fig. 2a). Such phenotypes were observed for 89.5% of calli, and the frequency of completely white only calli was 49% (Fig. 2c). Similar frequencies were observed after transformation with the maize optimized-codon zCas9 gene. Neither the frequency of white callus nor the frequency of combined white and mosaic calli differed statistically from those observed for AteCas9 calli (*p* = 0.198 and 0.293, respectively). However, significantly lower frequency of completely white callus (14.8%, *p* < 0.01) was observed for transformants containing the plant codon-optimized Cas9p variant. As the frequency of mosaic callus was similar for all three Cas9 variants used, the combined frequency of white and mosaic callus using Cas9p (64.8%) was also lower than AteCas9 (*p* = 0.025) and zCas9 (*p* = 0.002).

### Variation in color among transgenic calli results from repressed anthocyanin accumulation

**Fig. 3:**
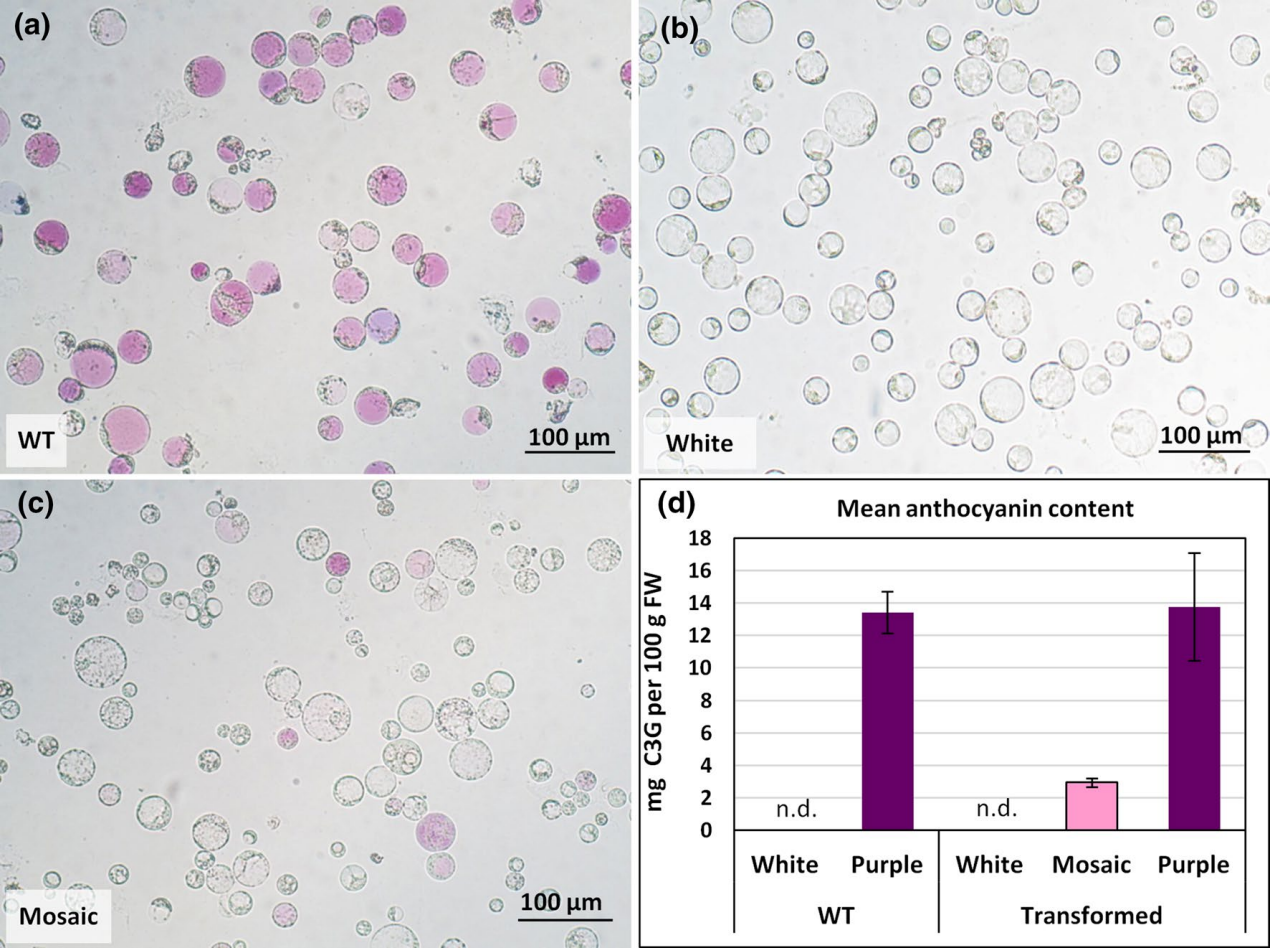
Microscopic and quantitative evaluation of anthocyanin pigments loss in transgenic callus. **a** In WT callus, the main fraction was consisted of intense purple-colored protoplasts, while white protoplasts are occasionally observed. **b** Only white protoplasts were present in transgenic white callus. **c** Fraction of completely white protoplasts dominates in transgenic mosaic callus and purple protoplasts are faint. **d** Mean anthocyanin content in WT and transgenic callus of various colors expressed in mg cyanidin-3-glucoside (C3G) equivalent per 100 g fresh callus weight as determined spectrophotometrically. Whiskers—standard error of the mean; n.d.—anthocyanins were not detected

Color differences in transgenic callus may likely result from either lower accumulation of anthocyanins in all cells or repressed accumulation in only some of them. To reveal reasons for observed variation, we measured anthocyanin content in callus and then screened these callus tissues at the cell level. The obtained calli were grouped to phenotypically different classes based on color, i.e., deep purple, completely white, and mosaic regardless, which Cas9 vector was used for transformation. All protoplasts released from WT white calli and observed under the microscope were transparent white, while protoplasts of WT purple calli were mainly purple, and those of intense hue dominated. Protoplasts with faint color were rare, though a small fraction of completely white protoplasts was observed (Table 2; Fig. 3a). All protoplasts released from white transgenic calli were completely free of pigmentation (Fig. 3b). Mosaic calli were heterogeneous and the mean frequency of purple protoplasts was only 25.8% (Fig. 3c). Hue of most transgenic purple protoplasts was usually less intense than that of protoplasts from WT purple calli, and differentiation between very faint and completely white protoplasts was not possible in this experiment. White protoplasts were also observed in transformed purple calli and their frequency (27%) was significantly higher (*p* < 0.001) than in WT purple calli (Table 2). Taken together, these discoloring phenotype results from protoplasts are consistent with discoloring phenotype of callus clumps that we examined earlier (Fig. 2).

**Table 2:** Mean number and frequency of white protoplasts in WT and transgenic calli

Callus color	Number of protoplasts		Frequency of white protoplasts (%)	*χ* ^2^
	Total	White		
Purple (WT)	524	32	6.1	–
Purple	725	196	27.0	62.8*
Mosaic	1741	1292	74.2	272.4*
White	572	572	100.0	317.8*

*Significant difference between the frequency of white protoplasts in transgenic and WT calli at *p* < 0.001

We then tried to measure anthocyanin content to validate the discoloring phenotype which is due to loss of anthocyanin pigments. Purple WT calli contained high amounts of anthocyanins equivalent to 13.4 mg C3G per 100 g of fresh callus weight as determined spectrophotometrically (Fig. 3d). As a control, we used the white callus line from which the purple carrot line was derived. No anthocyanins were detected in cells of this white callus line. In transgenic white calli, no anthocyanins were detected, but in purple calli, the anthocyanin content was, on average, at the same level as in the purple WT calli (Fig. 3d). Mosaic calli had, on average, 78% less anthocyanins (2.9 mg C3G per 100 g FW) than WT purple calli that corresponded to a lower fraction (25.8%) of anthocyanin accumulating cells (93.9% in WT purple callus). Thus, the pigment content in calli is highly correlated with the frequency of purple protoplasts (*r* = − 0.89; *p* = 0.019).

These combined data indicate that callus discoloration after transformation with CRISPR/Cas9 vectors is, indeed, due to loss of anthocyanin accumulation, presumably due to targeted knockout of the *F3H* gene.

### Cas9 induces loss-of-function mutations in the target carrot *F3H* gene

To validate that callus discoloration was due to the occurrence of intended mutations at target sites of *F3H*, we performed molecular genotyping analysis. It is known that Cas9 usually generates DSBs at the third-nucleotide proximal to PAM in the target sequence, which is the hotspot for mutation occurrence. In our design, the gRNA4 sequence contains a restriction site located proximal to PAM and recognized by the *Nco*I nuclease (Fig. 1b). We expected that successful targeting of *F3H* with gRNA4 should result in mutations that destroy the *Nco*I restriction site, which can be confirmed by the restriction fragment length polymorphism (RFLP) assay. We first performed a nested PCR to ensure correct amplification of the desired 538 bp-long *F3H* fragment (Fig. 1b) from carrot genome and then digested final PCR products with *Nco*I nuclease.

**Fig. 4:**
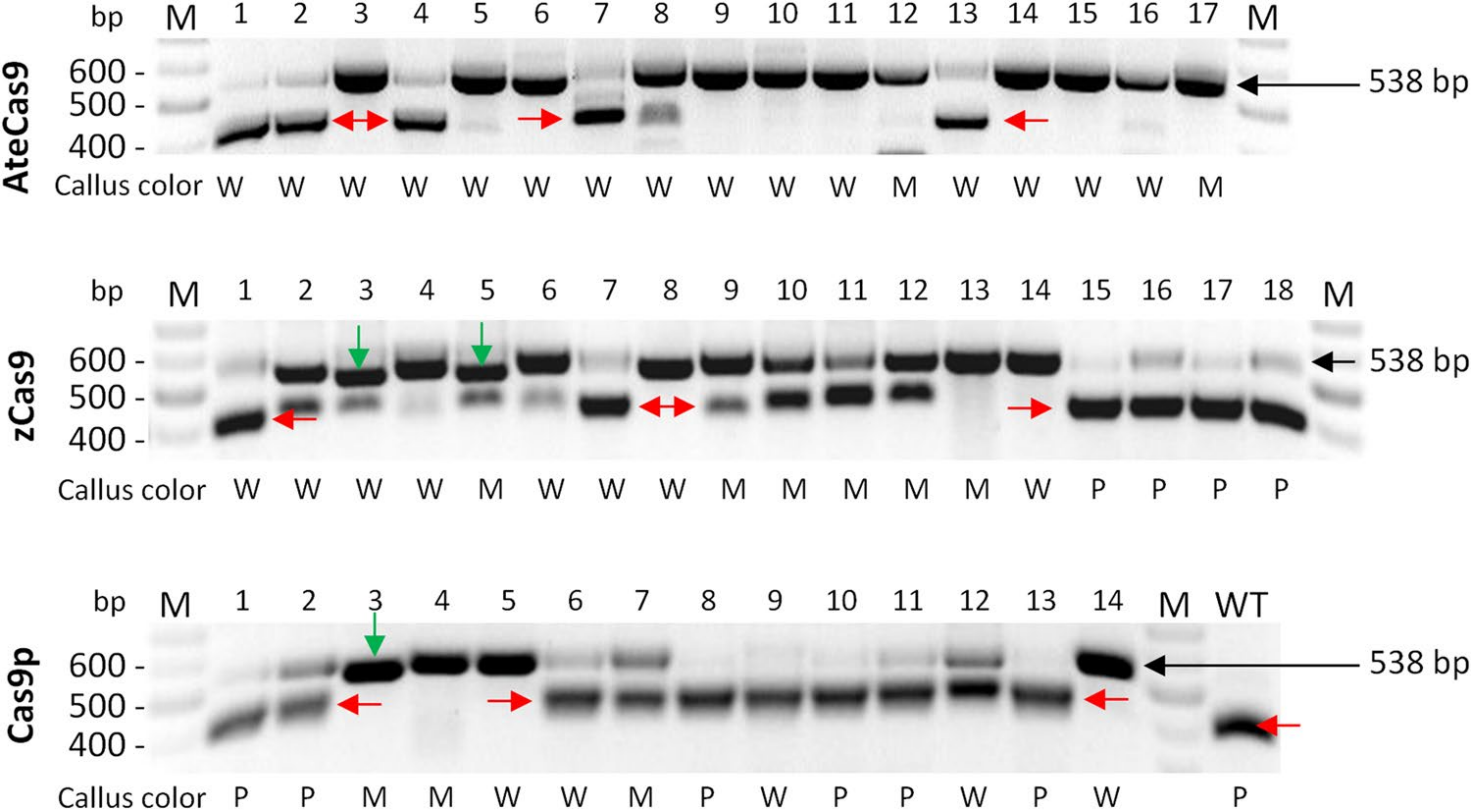
Genotyping Cas9-induced mutations by RFLP analysis. A nested PCR with F3H primers was carried out for individually selected callus transformed with one of three codon-optimized Cas9. Then, PCR products were digested with *Nco*I nuclease. The complete digestion leads to two expected fragments of 391 bp (red arrow) and 147 bp (not shown). When cleavage site is mutated, the expected length of the not digested product is 538 bp. Short deletions (18–25 bp) at the target site lead to restriction fragments of other lengths (green arrows). *WT* completely digested products when DNA was isolated from wild-type callus. *M* GeneRuler DNA Ladder Mix. Callus color: *P* purple, *M* mosaic, *W* white

As expected, amplicons derived after PCR with DNA of WT callus were completely digested resulting in two fragments of 391 and 147 bp (Fig. 4). In contrast, digestion of PCR products from transgenic calli resulted in either two DNA fragments, uncleaved DNA or a mixture of both cleaved and uncleaved DNAs (Fig. 4). The presence of uncleaved products for transgenic calli indicated that *Nco*I recognition site was mutated. Samples where *Nco*I did not digest PCR products usually originated from white transgenic calli and most frequently represented AteCas9 transformed calli. Samples with complete digestion predominantly represented Cas9p calli, consistent with the phenotypic data (Fig. 2). Calli transformed with AteCas9 resulted in most completely undigested PCR products. Although zCas9-expressing calli resulted in similar numbers of completely undigested products to Cas9p-experssing calli, they contain more partly undigested products than Cas9p samples (Fig. 4). These results again suggest that AteCas9 is most efficient in carrot cells for generating mutations, followed by zCas9 and Cas9p. Independent on the Cas9 variant used, undigested products were also present in both purple and mosaic callus, indicating that at least some cells were mutated (e.g., samples 5 or 15 from the second gel in Fig. 4).

**Fig. 5:**
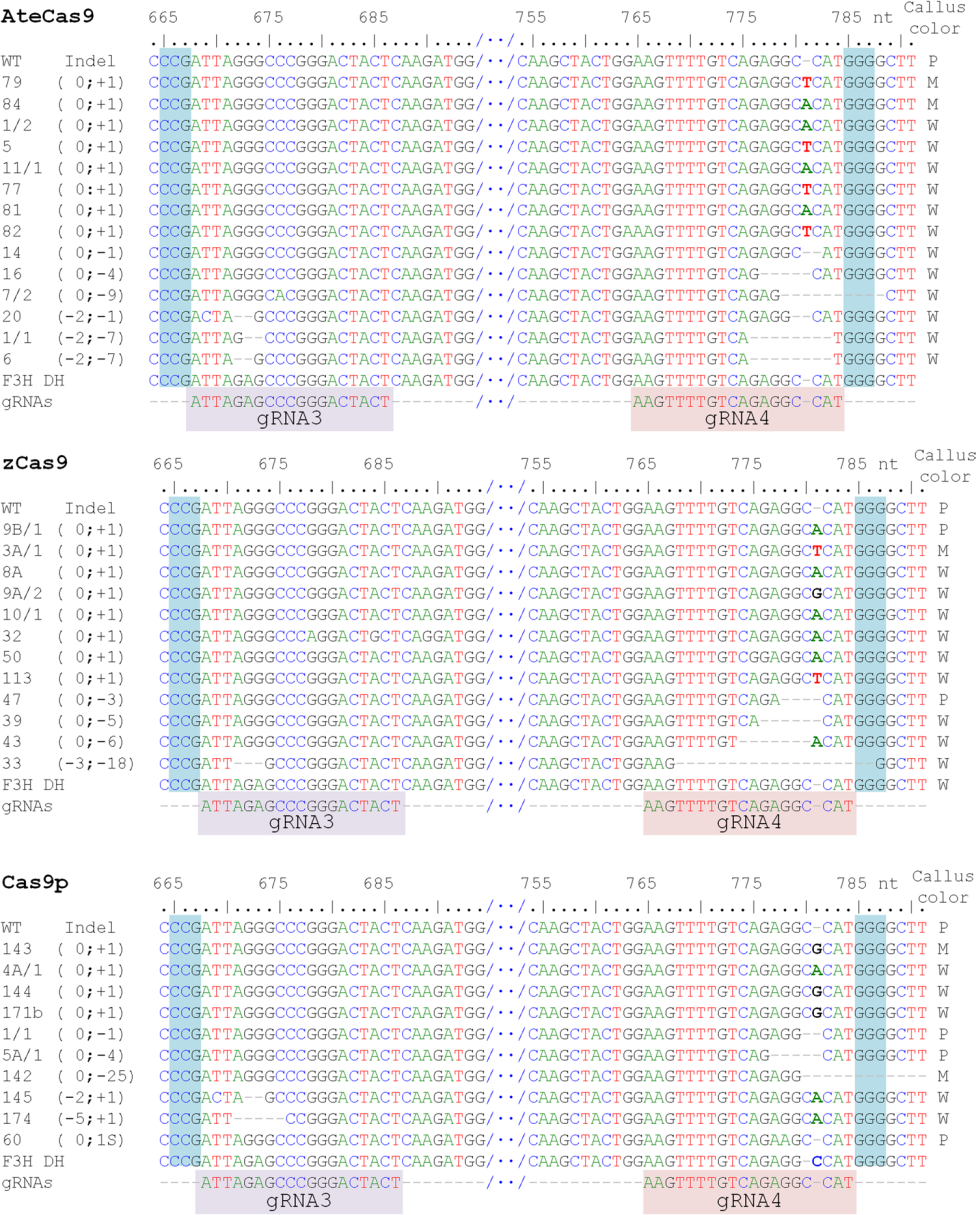
Genotyping Cas9-induced mutations in the *F3H* gene by sequencing. Targeted sites by gRNA3 and gRNA4 are shown only. Sequences are aligned to the *F3H* sequence fragments from wild-type purple callus. The number of inserted or deleted nucleotides at gRNA3 and gRNA4 targeted sites, respectively, is shown in parentheses. *WT* sequence of *F3H* gene fragments from wild-type purple callus, *F3H DH* reference sequence of the *F3H* gene (NCBI XM_017385173). PAM sequence is shown with color background. Callus color: *P* purple, *M* mosaic, *W* white

To confirm that the *Nco*I restriction site was, indeed, mutated, we sequenced products of the expected 538 bp length and those which had larger deletions. The results revealed mutations that destroy *Nco*I recognition site in calli transformed by all three Cas9 variants (Fig. 5). The most frequent changes were single-nucleotide insertions, mainly A or T, between the third- and fourth-nucleotide upstream the PAM. Short deletions up to 18 nt were the second most frequent type of mutations. In all calli, deletions usually occurred upstream PAM, but there were also deletions, terminating or eliminating the PAM sequence (Fig. 5).

### Single-nucleotide mismatch in the gRNA seed region lowers frequency but does not prevent Cas9 cleavage

**Fig. 6:**
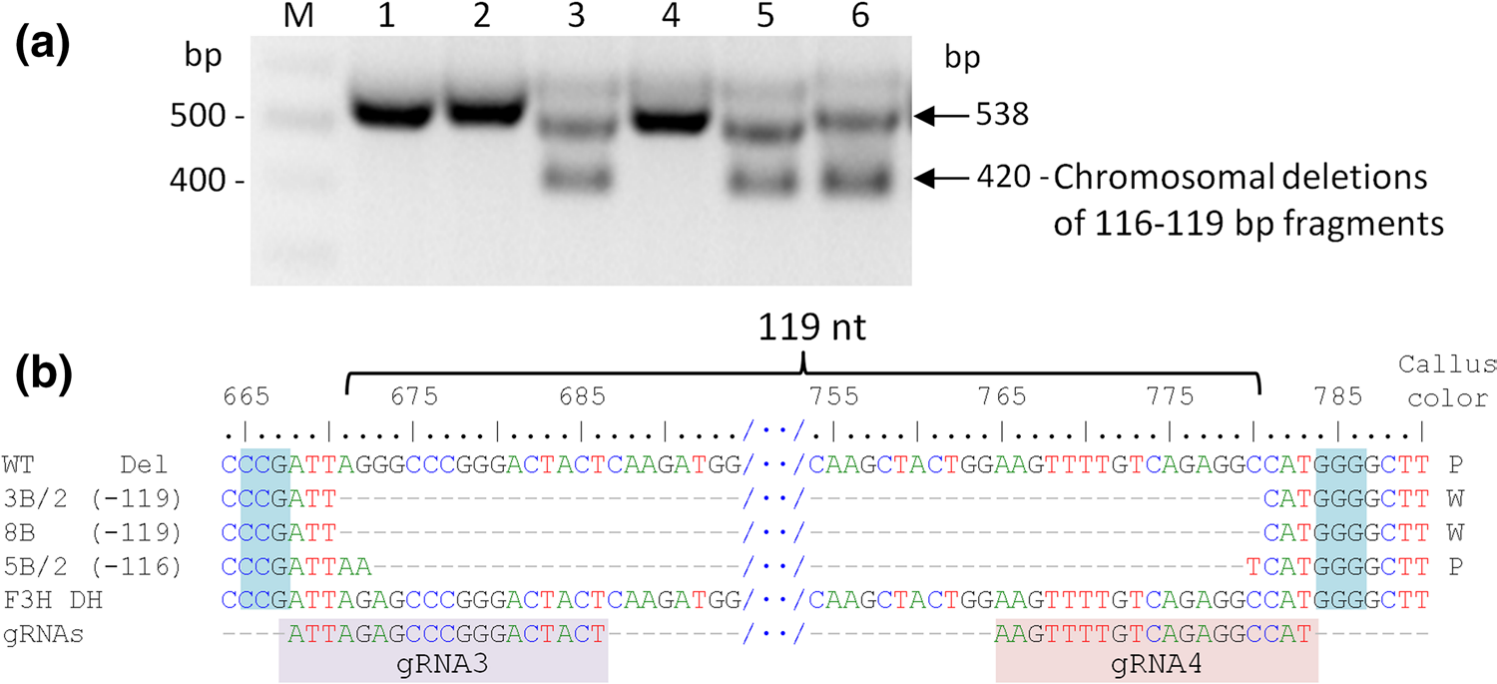
Chromosomal deletions generated by two DSBs. Deletions of 116–119 nt between gRNA3 and gRNA4 target sites in the *F3H* gene detected by PCR and by sequencing of these products. Sequences are aligned to the *F3H* sequence fragments from wild-type purple callus. The number of deleted nucleotides between gRNA3 and gRNA4 targeted sites is shown in parentheses. *WT* sequence of *F3H* gene fragments from wild-type purple callus, *F3H DH* reference sequence of the F3H gene (NCBI XM_017385173). PAM sequence is shown with color background. Callus color: *P* purple, *M* mosaic, *W* white. **a** Detection of large chromosomal deletions in some callus samples (e.g., #3, 5, and 6). **b** Sequences of *F3H* fragments with chromosomal 116–119 nt-long deletions

It is well known that CRISPR/Cas9 may result in off-targeting at unintended sites which share sequence homology with the target site (Fu et al. 2013, Hsu et al. 2013). In this study, we used two gRNAs for simultaneous targeting of *F3H*. The second gRNA (i.e., gRNA3) was originally designed using the reference *F3H* gene of DH1 carrot. Sequencing of *F3H* fragments from WT purple callus used in our study revealed that gRNA3 had a A/G mismatch at the sixth-nucleotide upstream the PAM which is within a seed region crucial for Cas9/gRNA binding. Despite this mismatch, a quarter of sequences mutated at the *Nco*I site at the gRNA4 target had additional mutations at the gRNA3 target (Fig. 5). These mutations were detected with all three Cas9 versions. Given the relatively high off-targeting activity of gRNA3, these multiplexing Cas9 constructs should result in large deletions due to simultaneous cleavage at both gRNA3 and gRNA4 target sites. We, indeed, identified such large deletions in transformed calli (Fig. 6a). Sequencing of these products confirmed deletions of 116–119 nt in length (Fig. 6b). The deleted chromosomal region encompassed gRNA3 and gRNA4 target sequences and the whole fragment spanning between them. Two of these chromosomal deletions (− 119 nt) were canonical with three nucleotides proximal to PAM left at both target sites, representing perfecting ligation after Cas9 cleavage at both sites. These results suggest that CRISPR/Cas9 can tolerate 1 nt mismatch even in the seed sequence of the protospacer in carrot cells.

## Discussion

Callus purple pigmentation provides evidence that anthocyanin pathway genes are functional in this tissue, although in carrot plants, anthocyanin biosynthesis is tissue specific and developmentally regulated (Xu et al. 2014). We measured anthocyanin contents in callus and found that their amounts were high, and comparable to levels in purple roots of other carrot varieties (Alasalvar et al. 2005; Li et al. 2012). Moreover, purple calli used here were almost homogeneous with regard to pigmentation as most cells (93%) exhibited deep purple color visible under a microscope. These findings supported the decision to use purple callus as a convenient model for validating CRISPR/Cas9 systems in carrot by targeting a gene in the anthocyanin biosynthesis pathway. The *F3H* gene was shown earlier to be critical for anthocyanin biosynthesis in some species, and thus, knockout of this gene will abolish anthocyanin accumulation (Bowerman et al. 2012). We, therefore, targeted *F3H* gene in purple callus to generate its knockouts using three Cas9 variants as, to our best knowledge, Cas9 has not been tested and optimized in carrot till now.

The developing white cell aggregates on a surface of explant callus after Cas9 constructs delivery were clearly distinguished from purple callus mass exposed to the selection medium. The occurrence of white or faint purple or mosaic calli indicated that a repression of anthocyanin biosynthesis had occurred as a result of successful site-directed mutagenesis in the targeted *F3H* gene. Mosaic tissue composed of white and purple cells occurred at high frequency. As callus explant was used for *Agrobacterium*-mediated transformation, it was expected that closely localized transgenic cells could aggregate during the selection, resulting in a heterogeneous tissue composed of mutated and not mutated cells. High frequency of heterogeneous tissue was also obtained when Cas9-mediated genome editing was tested in tobacco BY2 suspension cells as shown by Mercx et al. (2016). The authors suggested that coexistence of mutated and WT cells in developing calli could be a result of a delayed Cas9 cleavage occurring after the first division of the transformed cell, which is also a possible scenario in our work.

Our RFLP assay at the gRNA4 target site, along with Sanger sequencing validation, provided molecular evidence for intended mutations in the *F3H* sequence. Hence, we successfully demonstrated CRISPR/Cas9-based genome editing in carrot cells. Moreover, our CRISPR/Cas9-based reverse genetic approach confirmed the critical role of the *F3H* gene in anthocyanin biosynthesis in carrot. Furthermore, a close comparison of three Cas9 codon-optimized versions suggests that the AteCas9 system is most efficient in generating targeted mutations in our system. This AteCas9 was codon-optimized for *Arabidopsis* (Fauser et al. 2014; Schiml et al. 2014), which is a dicot plant species just like carrot. We fixed the gRNA system and also used the same promoter to drive expression of all three Cas9 in the same T-DNA backbone. Therefore, the observed differences in mutation efficiencies must be directly related to Cas9 sequences. However, these three Cas9 sequences vary in codon-optimization, N or C-terminus modification, and position and sequence of nucleus localization signals (NLS). Any of these factors can play a role on impacting Cas9 activity.

Sanger sequencing revealed that the mutations are mainly single-nucleotide insertions or short deletions, and most of these mutations will probable cause gene knockout due to introduction of early stop codon as well as changes of coding sequences. In carrot calli transformed with AteCas9 and zCas9 constructs, insertions occurred twice more often than deletions, but among Cas9p transgenic calli, a 1:1 ratio of insertions to deletions was found, similarly as shown in rice genome editing studies (Xie and Yang 2013). We observed that A or T insertion are the most common types of insertions, which were consistent with an early report for CRISPR-Cas9 system (Ito et al. 2015).

A multiplexing gRNA approach in designing CRISPR/Cas9 vectors increases the probability of mutations within the target gene. The frequency of mutations induced by different gRNAs depends on several factors, but three scenarios can be considered, assuming that only two gRNAs are used: (1) only one of two targeted sites is mutated, (2) both sites are mutated independently, and (3) simultaneous DNA cleavage at both sites led to the excision of the whole fragment between targets. Although the occurrence of the later is less frequent, deletion of a large fragment increases the probability of knocking out the target gene (Johnson et al. 2015). This approach was successfully demonstrated in *Arabidopsis* by designing two gRNAs targeting sequences separated by a 48 bp fragment (Li et al. 2013) or in tomato where targeted sites were in 140 bp distance (Brooks et al. 2014). In contrast, the elimination of 218 bp sequence between two gRNA targets in potato was not identified (Butler et al. 2015). We simultaneously targeted two *F3H* sites located in the 125 bp distance and we identified transgenic calli with 116–119 bp chromosomal deletions as predicted. Targeting such a large fragment for deletions with CRISPR/Cas9 facilitates a molecular identification of edited events in routine screening, as shorter PCR products can be easily distinguished during electrophoresis.

In this study, we found a prominent level of tolerance with a single-nucleotide mismatch at the seed sequence of the protospacer of gRNA3. Earlier reports have indicated that the presence of an unpaired nucleotide in the gRNA seed (core) region makes CRISPR/Cas9 genome editing impossible as the seed region is critical for correct binding of a Cas9/gRNA complex to the target DNA (Hsu et al. 2013; Xie and Yang 2013). Recent studies have shown that some mismatches in the seed region are tolerated; however, they markedly lower the activity of such Cas9/gRNA complex (Andersson et al. 2017). The tolerance of mismatches has been confirmed in human cells (Pattanayak et al. 2013), but also in rice (Zhang et al. 2014) and sweet orange (Jia and Wang 2014). Our result suggests that it is critical to choose unique target sequence to ensure precise genome editing with minimal off-target effects. Many design tools are available for use in plants (Liu et al. 2017; Xie et al. 2017). Off-targeting can be mitigated using pair-nickases of Cas9 (Ran et al. 2013; Fauser et al. 2014), high-fidelity Cas9 variants (Slaymaker et al. 2016), or their hybrid (Kulcsár et al. 2017). Recently, we developed a highly efficient CRISPR/Cpf1 system in rice with high specificity (Tang et al. 2017). In the future, it will be interesting and useful to further test and apply these systems in carrot for precise genome editing.

In conclusion, we show, for the first time, successful site-directed editing of carrot genome by the delivery of the CRISPR/Cas9 system. This was demonstrated by generating knockout mutations in the *F3H* gene, which is critical for anthocyanin biosynthesis. Mutations induced by Cas9/gRNA complexes resulted in suppression of anthocynin accumulation, rendering easy visual identification of knockout events. A time-efficient protocol of *A. tumefaciens*-mediated transformation of carrot callus, easy selection, and phenotypic changes of edited cells make purple callus a suitable model for developing and testing genome editing reagents. We used this model to show different efficiency of three SpCas9 variants for targeted mutagenesis in carrots, with AteCas9 reaching up to 90% efficiency. This simple and fast screening model can be valuable for comparison purposes, validation new CRISPR editing systems, or testing various delivery approaches, including ribonucleoprotein complexes.

### **Author contribution statement**

MK-Ch, RB, and YQ designed the experiment, MK-Ch, TO, and LGL performed the experiments. MK-Ch, TO and RB analyzed the results, MK-Ch, RB, and YQ wrote the manuscript. All authors read and approved the manuscript.

## Electronic supplementary material

Below is the link to the electronic supplementary material.

**Fig. S1** Confirmation of transgenic calli by PCR. Amplified products in PCR with primers specific to the *aph* gene and to the fragment comprising the 35S promoter and *Cas9* gene. The product of 398 bp is expected for *aph* gene after amplification with CF3 and Cas9-R primer pair. Two products in the range between 576–695 bp and 903–1022 bp are expected for *SpCas9* genes, depending on the SpCas9 variant used, after amplification with 35S-Cf3 and Cas9-R primer pair. PCR was set up on plasmid DNA harboring different *SpCas9* genes (lanes: 1—pAteCas9, 2—pzCas9, and 3—pCas9p) or using DNA of transgenic callus (lanes: 4—6 AteCas9; 7—9 zCas9; 10—12 Cas9p). NTC—no DNA template control, WT—DNA isolated from wild-type purple callus. M—GeneRuler DNA Ladder Mix (TIF 339 KB)
