# Supplementary figures and images for: Efficient CRISPR/Cas9-based genome editing in carrot cells
Source: Plant Cell Rep. 2018 Jan 13;37(4):575–86. doi: 10.1007/s00299-018-2252-2 (PMC5852178; doi:10.1007/s00299-018-2252-2)

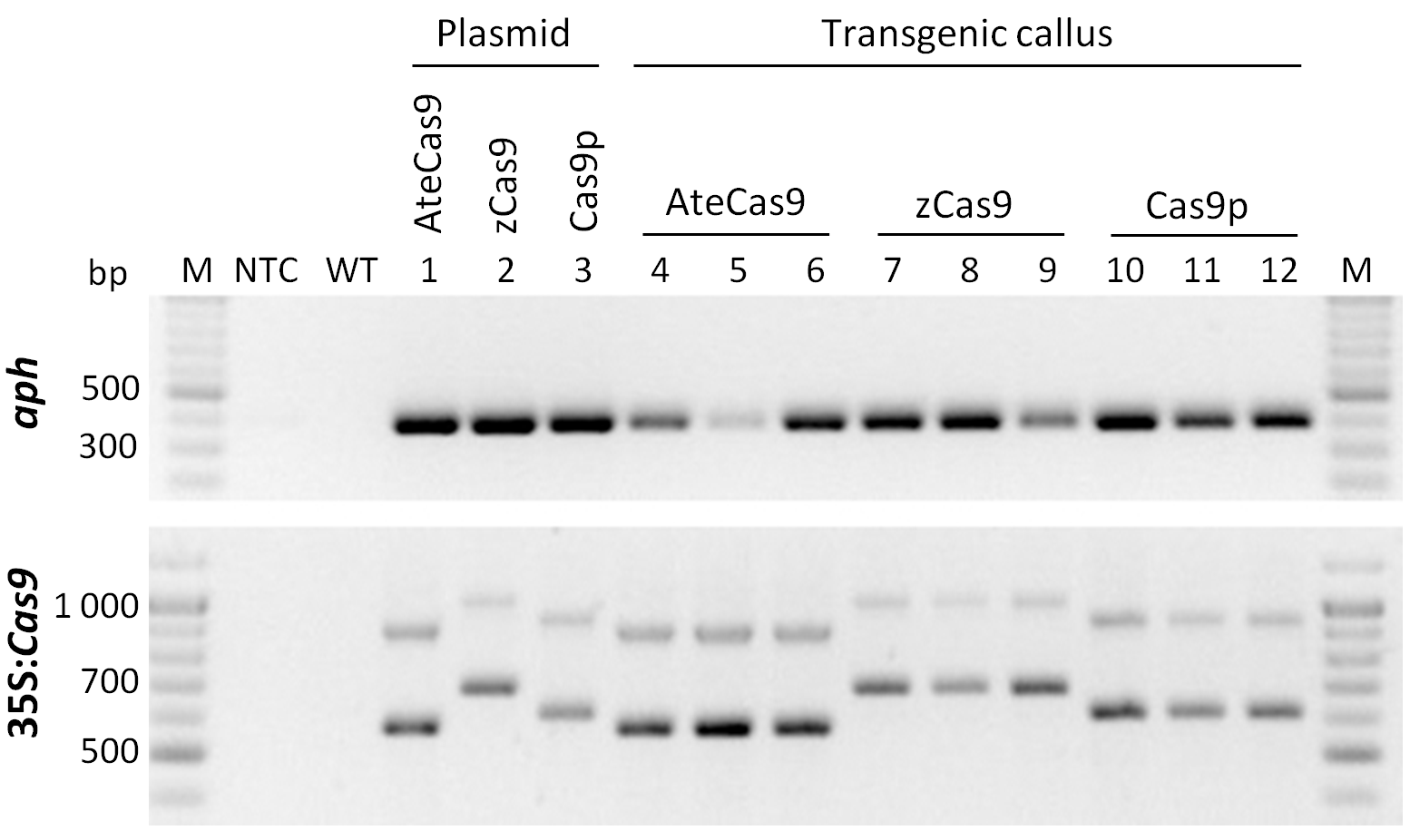

Supplement: Supplementary file 1 — Fig. S1 Confirmation of transgenic calli by PCR. Amplified products in PCR with primers specific to the aph gene and to the fragment comprising the 35S promoter and Cas9 gene. The product of 398 bp is expected for aph gene after amplification with CF3 and Cas9-R primer pair. Two products in the range between 576–695 bp and 903–1022 bp are expected for SpCas9 genes, depending on the SpCas9 variant used, after amplification with 35S-Cf3 and Cas9-R primer pair. PCR was set up on plasmid DNA harboring different SpCas9 genes (lanes: 1—pAteCas9, 2—pzCas9, and 3—pCas9p) or using DNA of transgenic callus (lanes: 4—6 AteCas9; 7—9 zCas9; 10—12 Cas9p). NTC—no DNA template control, WT—DNA isolated from wild-type purple callus. M—GeneRuler DNA Ladder Mix (TIF 339 KB) [file 299_2018_2252_MOESM1_ESM.tif]
